# Clinical profiles of adolescent personality pathology: a latent structure examination of the Semi-Structured Interview for Personality Functioning DSM-5 (STiP-5.1) in a help-seeking sample

**DOI:** 10.1186/s40479-024-00252-5

**Published:** 2024-04-09

**Authors:** Madelyn Thomson, Marialuisa Cavelti, Stefan Lerch, Julian Koenig, Corinna Reichl, Ines Mürner-Lavanchy, Andrea Wyssen, Michael Kaess

**Affiliations:** 1 University Hospital of Child and Adolescent Psychiatry and Psychotherapy, University of Bern, Bern, Switzerland; 2 Graduate School for Health Sciences, University of Bern, Bern, Switzerland; 3 Department of Child and Adolescent Psychiatry, Psychosomatics and Psychotherapy Faculty of Medicine, University of Cologne, University Hospital Cologne, Cologne, Germany; 4 Department of Child and Adolescent Psychiatry, Centre for Psychosocial Medicine, University of Heidelberg, Heidelberg, Germany

**Keywords:** Personality pathology, Adolescence, Clinical heterogeneity, Dimensional models, Latent structure

## Abstract

**Background:**

Despite the introduction of dimensional conceptualisations of personality functioning in the latest classification systems, such as Criterion A of the Alternative Model of Personality Disorders in the *DSM-5*, heterogeneous clinical presentation of personality pathology remains a challenge. Relatedly, the latent structure of personality pathology as assessed by the Semi-Structured Interview for Personality Functioning DSM-5 (STiP-5.1) has not yet been comprehensively examined in adolescents. Therefore, this study aimed to examine the latent structure of the STiP-5.1, and, based on those findings, to describe any unique clinical profiles that might emerge.

**Methods:**

The final sample comprised 502 participants aged 11–18 years consecutively recruited from a specialised personality disorder outpatient service, as well as general day clinic and inpatient wards at the University Hospital University Hospital of Child and Adolescent Psychiatry and Psychotherapy, University Hospital Bern, Switzerland. Participants were assessed using the STiP-5.1, as well as a battery of other psychological measures by clinical psychologists or trained doctoral students. Variations of Factor Analysis, Latent Class Analysis and Factor Mixture Models (FMM) were applied to the STiP-5.1 to determine the most appropriate structure.

**Results:**

The best fitting model was an FMM comprising four-classes and two factors (corresponding to self- and interpersonal-functioning). The classes differed in both overall severity of personality functioning impairment, and in their scores and clinical relevance on each element of the STiP-5.1. When compared to the overall sample, classes differed in their unique clinical presentation: class 1 had low impairment, class 2 had impairments primarily in self-functioning with high depressivity, class 3 had mixed levels of impairment with emerging problems in identity and empathy, and class 4 had severe overall personality functioning impairment.

**Conclusions:**

A complex model incorporating both dimensional and categorical components most adequately describes the latent structure of the STiP-5.1 in our adolescent sample. We conclude that Criterion A provides clinically useful information beyond severity (as a dimensional continuum) alone, and that the hybrid model found for personality functioning in our sample warrants further attention. Findings can help to parse out clinical heterogeneity in personality pathology in adolescents, and help to inform early identification and intervention efforts.

**Supplementary Information:**

The online version contains supplementary material available at 10.1186/s40479-024-00252-5.

## Background

The perennial debate concerning the underlying or latent structure of personality pathology as either distinct categories or as a dimensional continuum of psychopathology [1, 2] has gained increased traction since the inception of the *Diagnostic and Statistical Manual of Mental Disorders, Fifth Edition* [3], and persists with the recent release of the *International Classification of Diseases, 11th version* [4]. Correspondingly, the overall conceptualization and classification of personality disorders (PD) are undergoing a significant departure from categorical diagnoses toward dimensional approaches. This is a result of efforts to overcome well-documented shortcomings of traditional categorical models of PD including limited diagnostic reliability, diagnostic overlap between PD, within-category heterogeneity, arbitrary thresholds, and more [5, 6].

Criterion A from the Alternative Model of PD (AMPD) in Section III of the *DSM–5* [3] is one example of the dimensional conceptualization of PD, and defines the presence and overall severity of PD by the degree of impairment in self- and interpersonal-functioning [3]. Self- and interpersonal-functioning deficits are considered core features of personality pathology, in that they have been found to distinguish not only between those who do and do not have PD, but also between personality pathology and other types of psychopathology [7–9]. Research also indicates that Criterion A predicts a number of negative outcomes over and beyond traditional PD categories including current or future adjustment [10–13], accounts for the comorbidity among categorical PD diagnoses [14], is sensitive to change [15] and is deemed to have overall enhanced clinical utility [16].

As Criterion A centres around development of self [17] – a particularly important task of adolescent development – it is posited to be more developmentally sensitive in the assessment of personality pathology in adolescents [18]. Indeed, emerging evidence on a clinician-rated interview for Criterion A, the Semi-Structured interview for Personality Functioning DSM-5 (STiP-5.1) [19], suggests that the STiP-5.1 in particular might be more suitable for the detection of the severity of personality pathology in adolescents than in adults, and distinguishes normative adolescent difficulties from personality pathology [20]. Furthermore, adolescence is a developmentally sensitive period for PD development, and is considered a critical window for early detection and intervention of PD [21–23]. Therefore, as Criterion A is gaining importance as a focus for intervention and treatment [9], it might also prove to be clinically important in facilitating the early detection of personality pathology, and more informative for planning treatment for PD in adolescents [20].

However, whilst the introduction of Criterion A presumably overcomes the pervasive problem of overlap between PD diagnoses and heterogeneity within the discrete categories, the clinical presentation of patients based on the dimensional Criterion A might still be expected to have many variants, as some heterogeneity persists even in dimensional measures of psychiatric disorders [24]. This is particularly relevant for personality functioning reflected in Criterion A, as although it is designed to indicate an overall score of severity on a continuum, it is a multifaceted construct covering two separate domains, four elements, and 12 facets which can each be scored from ‘little to no impairment’ to ‘extreme’ impairment in functioning. This presents a challenge for clinicians and researchers alike regarding understanding the aetiology of the disorder, identification, diagnosis, and ultimately, treatment. Therefore, although dimensional assessment provides useful clinical information regarding severity, finding homogenous groups within this complex construct should aid in clinical utility. Efforts then, to enhance understanding and parse out heterogeneity in personality pathology reasonably leads to the examination of the underlying latent structure.

Although various methods for examining the latent structure of a concept can be used, typically two types of analytical approaches are adopted in the investigation of the latent structure of PD, which can be broadly categorized as 'variable-centred' and 'person-centred,' corresponding to two approaches exploring whether a structure is best represented as dimensional or categorical. Factor Analysis (FA) operates on the notion that variables can be reduced to fewer underlying latent factors (variable-centred) that share common variance, investigating the common content among items. Individuals are thought to differ from each other according to their scores on the underlying latent factors [25], and therefore presupposes a difference in *degree* – a dimensional approach. A recent study used FA to examine the latent structure of the STiP-5.1 in adults, with results supporting a two-factor structure reflecting self- and interpersonal-functioning [26].

Latent class analysis (LCA), on the other hand, works on an individual level (person-centred) and aims to find heterogeneity in a population, whereby individuals are classified according to patterns of scores into subtypes that are thought to be homogeneous subgroups of the disorder. That is, finding “hidden groups,” presupposing a difference in *kind* – or a categorical approach – in this case, of personality functioning [27, 28]. The use of LCA in PD research has largely been limited to categorical Borderline Personality Disorder (BPD) [29–36] or adult samples.

More recent statistical advancements however, eliminate the requirement to choose between dichotomous conceptualizations. Factor Mixture Models (FMM) incorporate aspects of both LCA and FA, allowing for the underlying structure to be both categorical and dimensional [37]. This is done by allowing classification of individuals into subgroups (or classes), whilst simultaneously accounting for within-class heterogeneity by one or more latent factors (i.e., continuous variables such as severity) [38]. As with LCA, studies using FMM in PD have been mostly restricted to categorical BPD [30, 39–41] and adult populations.

As indicated above, efforts at elucidating meaningful clusters or subgroups in personality pathology research have largely focused on BPD as a categorical disorder, mostly with 3-cluster solutions found to be the best fit [42–45], along a severity dimension [29, 31, 32, 34, 35, 46]. For a more detailed overview, see eText 1 in Supplementary Materials. To our knowledge, only one study has explored classes of personality pathology (rather than ‘types’) based on the AMPD/ICD-11 conceptualisation of PD. They found 4 distinct profiles using latent profile analysis, with profiles reflecting both differences in severity, and also qualitatively. This was again restricted to BPD (and thereby incorporating Criterion B), in adults [47]. Direct comparison of FA, LCA, and FMM to determine the best structure of PD has also been limited to BPD, and there is only one study that has directly compared the three approaches in BPD criteria in adolescents [30]. They found a complex 2-class, one-factor FMM (a ‘borderline group’ and an ‘impulsive group’) to be the best fit [30].

To date, no studies have examined Criterion A as a broader dimensional measure of personality pathology using FMM in adolescents. Therefore, the current study had two main aims:

1. To explore the latent structure of the STiP-5.1 by comparing various models to determine the best model fit for personality functioning (Criterion A) in a sample of adolescents, as reflected in this measure.
2. Based on the first set of findings, to describe any distinct profiles that might emerge within the Criterion A construct on the basis of the STiP-5.1, and further characterise them with other clinical variables.

This study was exploratory (given that the STiP-5.1 has not yet been examined extensively in adolescent populations, particularly in the application of various models), and therefore, the identification of any clinical profiles was based only upon whether such profiles could be yielded in the first step.

## Methods

### Participants and procedures

Data for the current study were pooled across two separate studies. As consecutive recruitment for the two studies is ongoing, participant data for the current sample were collected between November 2018 and March 2022. Participants were consecutively recruited from the University Hospital of Child and Adolescent Psychiatry and Psychotherapy, University Hospital Bern, Switzerland, including both general psychiatric inpatient and day-care services (Bernese Basic Documentation (BeBaDoc) study sample), and a specialized PD outpatient service (*“Ambulanz für Risikoverhalten und Selbstschädigung”* (AtR!Sk) study sample). The latter provides low threshold initial contact, comprehensive diagnostic assessment of PD features, and evidence-based therapeutic intervention for adolescents where indicated. Inclusion criteria were: 11–18 years of age (inclusive; BeBaDoc sample), 12–17 years of age (inclusive; AtR!Sk sample), and sufficient fluency in German language skills. Exclusion criteria were: patients lacking capacity to understand study details or provide informed consent. Written informed consent was obtained from all participants, as well as from a parent or legal guardian for those under the age of 14 years. Participants were administered the STiP-5.1 [19], and other measures, as part of a larger battery of assessments conducted by highly trained doctoral students (BeBaDoc sample) or clinical psychologists (AtR!Sk sample). In the AtR!Sk study, assessments formed part of the routine diagnostic assessment procedures at entry to the clinic and therefore participants were not financially reimbursed. BeBaDoc participants received the equivalent of 20CHF worth of vouchers. The studies were conducted in accordance with the Declaration of Helsinki [48] and approved by the local ethics committee (BeBaDoc ethics ID: 2018–01339; and AtR!Sk ethics ID: 2018–00942).

### Measures

German versions of all measures were used in this study.

#### Demographic data

Information was collected using a standardised set of interview questions assessing age, sex, family and living situation, and school type.

#### Personality functioning

Personality functioning was assessed using the STiP-5.1 [19] which measures the severity of personality impairment reflected in the Level of Personality Functioning Scale (LPFS) of the AMPD, covering self- and interpersonal-functioning. These comprise four elements (identity, self-direction, empathy, and intimacy) of 3 facets each, for a total of 12 facets. For each of these, the level of functioning can be determined based on five levels ranging from little to no impairment (0), mild [1], moderate [2], severe (3) or extreme (4) impairment in functioning, and therefore, higher scores indicate higher levels of personality impairment. The STiP-5.1 has good psychometric properties including high internal consistency (Cronbach’s α=0.97), high inter-rater reliability (interclass correlations (ICC) ranging from 0.81 to 0.92), good construct validity, and shown to distinguish between ‘normal’ and ‘clinical’ respondents, as well as between those with and without PD [19]. The German version used in the current study also demonstrates good inter-rater reliability (ICC=0.93) [49]. Furthermore, it has also demonstrated good psychometric properties in adolescents [20]. For the present study, Criterion A Impairment in Personality Functioning was considered fulfilled according to *DSM-5* AMPD guidance. That is, an overall mean score of two (moderate impairment) is used as diagnostic threshold required for *general* PD, and for descriptive statistics purposes we also applied AMPD guidance for *trait-specified* and *specific* PD whereby the individual must score moderate or greater (two or higher) on two or more elements for a PD diagnosis, for broader clinical applicability. In line with this, we also considered clinically relevant thresholds for each individual element to be two or higher (moderate or greater).

#### Borderline personality disorder (BPD) symptoms

BPD criteria were assessed using the *Structured Clinical Interview for DSM-IV axis II Personality Disorders* (SCID-II-PD; [50]), which reflects categorical diagnostic criteria of BPD according to the *DSM-IV*, and remains unchanged in *DSM-5* Section II.

#### Psychiatric diagnoses

The *Mini International Neuropsychiatric Interview for Children and Adolescents* (MINI-KID; [51]) was used to assess current comorbid psychiatric disorders according to the *DSM-IV* and *ICD-10*. It is a brief standardized measure found to generate valid and reliable psychiatric diagnoses for children and adolescents [52].

#### Depressivity

*The Children’s Depression Rating Scale – Revised* (CDRS-R; [53]) is a semi-structured, clinician-rated interview comprising 17 items, and was used to assess severity of depressive symptoms. The CDRS-R encompasses cognitive, somatic, affective and psychomotor symptoms, with items rated on a scale of severity from 1–7 for 14 items, and 1–5 for three items. The German version used in the current study demonstrates good psychometric properties including high internal consistency [54].

#### Non-suicidal and suicidal behaviour

The *Self-Injurious Thoughts and Behaviors Interview* (German version; SITBI-G; [55]) was used to measure the occurrence and frequency of suicidal attempts, as well as the number of days with non-suicidal self-injury (NSSI), in the last year. The German version has demonstrated good psychometric properties [55].

#### Quality of life

*The Health-related quality of life questionnaire for children and young people and their parents –* KIDSCREEN-10 [56] was used to assess health-related quality of life (HRQoL). Responses are scored on a 5-point response scale (either from *‘not at all’* [1] to *‘extremely’* [5] or from *‘never’* [1] to *‘always’* [5] depending on the item) across 10 items, and demonstrates reliability and validity [56].

#### Emotion regulation

Difficulties in emotion regulation were assessed using the *Difficulties in Emotion Regulation Scale* (DERS-16; [57]). A short-version (16 items) of the original DERS, the DERS-16 is a self-report measure of emotion regulation difficulties which is theoretically driven and found to be psychometrically sound [57]. It is rated on a 5-point response scale from *‘almost never’* (1) to *‘almost always’* (5) with higher scores reflecting greater levels of emotion dysregulation.

### Statistical analyses

Statistical analyses were conducted over two main steps. In the first step, we identified the best fitting model by investigating the underlying latent structure of the STiP-5.1, comparing variations of LCA, FA and FMM. The comparison within and between latent model types (LCA, FA, and FMM), was guided by statistical criteria and conceptual considerations [58]. First, regarding statistical criteria, the Bayesian Information Criterion (BIC; [59]) was used for model comparison to determine the best fit. The BIC is considered to be stricter than other criteria [60], is computed as a function of the log likelihood with a penalty for model complexity [37, 58, 61], and is an approximate fit index where lower values indicate better fit [27]. In addition, for LCA and FMM, entropy was evaluated, which is a measure of the degree to which the latent classes are distinguishable, and the precision with which individuals can be placed into classes. Ranging from 0 to 1, higher values of entropy indicate clearer class separation. A value of ≥ 0.80 is recommended, when participants would be classified based on the ‘most likely class membership’ resulting from LCA or FMM for further analysis [62]. Entropy in our study was caculated by MPlus, which is based on a formula outlined by Ramaswamy and colleagues [63]. It should be noted that entropy was employed to identify problematic over extraction. Further, best fitting model selection is a known issue with mixture models in particular, and there is no commonly accepted methodology on how to compare models with differing numbers of classes (class enumeration; [37] and even different parametrizations. Therefore, while we chose the BIC for model comparison as it is a stricter measure and favours a more parsimonious solution, it might also be useful for the reader to view other fit indices across the models. To that end, we have additionally included alternative statistical fit indices for the tested models, for transparency purposes. Please see eTable 2 in Supplementary Materials. Second, regarding conceptual considerations, this was applied in the following four ways:

1. Determining the number of factors to test based on logical construction of the instrument (for FA);
2. The composition of each factor, in that they reflect the theoretical construction of the relevant factor (i.e., self-functioning facets in the self-functioning factor only);
3. Not iterating FMMs past FMM-3 due to increased complexity; and
4. Determining the number of classes or profiles (e.g. for LCA or FMM) for clinical utility.

For further detail regarding the conceptual considerations employed in the study, please refer to eText 2 in the Supplementary Materials.

We first modelled single-factor, two-factor, and four-factor confirmatory FA based on previous literature [26] and the proposed theoretical structure of the STiP-5.1 (i.e., unidimensional, two domains, four elements), using the BIC for statistical guidance of best comparative model evaluation. Next, we fit LCA models with an increasing number of classes. Due to the paucity of previous research using LCA on the LPFS/STiP-5.1, we took an exploratory approach and continued to fit models until the BIC and entropy were found to be acceptable. We then compared variations of FMM by fitting the FMM with increasing measurement invariance of one, two, or four factors, and up to six classes, dependent on when the BIC was lowest and then began to increase again (lower BIC value indicating a better fit). That is, for each FMM, three variations (i.e., FMM-1, FMM-2, and FMM-3) from the most to least restrictive models with decreasing measurement invariance (MI) (and therefore, increasing complexity) in each, were tested. MI demonstrates that a measurement has the same meaning to groups by assessing the psychometric equivalent of a construct across those groups [64]. MI can take many forms, in that parameters including factor loadings, factor means, factor covariances and item thresholds can be potentially different in each class [37], reflecting the added flexibility of FMM over other models. See eTable 1 in Supplementary Material for further detail on MI across FMM variants, and implications for interpretation. Once the best fitting FMM was chosen, it was compared with the best fitting LCA and FA models – again using the BIC (the lowest one being the better fit), entropy, and conceptual considerations – in order to determine the overall best fitting model (see Clark et al., 2013 for more detail on the overall approach to fitting the various FMMs, and model comparison).

In a second step, based on findings from the first step, we examined any resulting class profiles, and characterised each on relevant clinical variables. First, each class profile was described based on the mean of each element of the STiP-5.1, as well as whether or not each element met the clinically relevant threshold for personality impairment (i.e., a score of 2 or higher, indicating moderate or higher impairment). Second, patients were grouped according to their most likely latent class membership, and comparisons between each profile and the overall sample in individual STiP-5.1 elements and clinical variables were used to characterise the profiles. Clinical variables included: number of fulfilled BPD criteria, emotion regulation difficulties, depressivity, number of other psychiatric diagnoses, number of suicide attempts in the previous year, number of days with NSSI in the previous year, and HRQoL. As the data in the current sample were non-normally distributed, median scores were calculated, which are also considered to be less sensitive to outliers. Cut-offs as percentiles were then used to determine whether a variable score for each class profile was considered: i) within the average (within the 40th and 60th percentile); ii) above the average (above the 60th percentile); or iii) below the average (below the 40th percentile), when compared to the overall sample median. Extreme upper and lower scores were considered above the 70th or below the 30th percentiles, respectively. MPlus Version 8.6 [65]. was used for calculating the FA, LCA and FMM. All other analyses were conducted using STATA Version 17 [66].

## Results

### Participants

A total of 526 participants completed the assessments. However, 24 participants had missing data on the STiP-5.1 and were therefore excluded, leaving a final total sample of *N*=502. The mean age was 15.41 years (*SD*=1.53) and 397 (79.1%) participants were female. One hundred and thirty-eight participants (27.5%) met diagnostic threshold for Criterion A Impairment in Personality Functioning according to *DSM-5* AMPD guidance for broader clinical applicability of *trait-specified* and *specific* PD (i.e., 2 or higher on 2 or more elements). Further demographic and clinical descriptive statistics of the sample are presented in Table 1.

**Table 1:** Demographic and clinical characteristics of the sample (*N*=502)

Variable	M (SD) / n (%)
Living with biological parents	
Lives with both biological parents^a^ (n, %)	282 (56.2)
Family status	
Living together (n, %)	111 (45.3)
Parents separated/divorced (n, %)	127 (51.8)
Parents separated by death (n, %)	2 (0.8)
Parents never lived together (n, %)	4 (1.6)
Unknown/other (n, %)	1 (0.4)
School type (currently completing or graduated)^b^	
Primary School (ISCED levels 0–1; at least 6 school years) (n, %)	71 (14.1)
Secondary School (ISCED level 2; 9–10 school years) (n, %)	347 (69.1)
High School (ISCED level 3; 12–13 school years) (n, %)	80 (15.9)
Other (n, %)	4 (0.8)
STiP-5.1 scores M (SD)	
Total score	1.17 (0.72)
Identity	1.68 (0.95)
Self-direction	1.30 (0.96)
Empathy	0.85 (0.78)
Intimacy	0.87 (0.87)
Number of fulfilled BPD criteria (M (SD))	2.70 (2.29)
Depressivity (CDRS-R) (M (SD))	51.86 (16.38)
Emotion Dysregulation (DERS) (M (SD))	59.44 (13.35)
HRQoL (M (SD))	19.17 (6.69)
MINI-KID (number of diagnoses) (M (SD))	2.82 (2.27)
NSSI (in the past year) (M (SD))	57.34 (91.69)
Suicide attempts (in the past year) (M (SD))	3.61 (25.14)

*STiP-5.1* Semi-structured Interview for Personality Functioning DSM-5 higher scores (higher impairment), *BPD* Borderline Personality Disorder, *CDRS-R* Children’s Depression Rating Scale – Revised, *DERS* Difficulties in Emotion Regulation Scale, *HRQoL* Health Related Quality of Life, *MINI-KID* The Mini International Neuropsychiatric Interview for Children and Adolescents, *NSSI* Non-Suicidal Self-Injury

^a^Remaining participants who do not live with both biological parents might live with only one biological parent, step-parents, or any other variation of family composition regarding parents/legal guardians

^b^Education levels are based on International Standard Classification of Education (ISCED)

### Step 1: Comparison of FA, LCA and FMM for identification of the best fitting model

Table 2 presents the model comparison and fit indices for the various models of FA, LCA and FMM. First, we compared one, two and four factor models for FA, with the four-factor model (reflecting the four elements of the STiP-5.1) having the best fit (BIC=16,483.77). Next, we compared LCA models across one to nine classes, with the eight-class model having the best fit (BIC=16,402.64), and an entropy value of 0.90. We then compared FMMs and identified the FMM-3 four-class, two-factor (reflecting self- and interpersonal-functioning) as the best fitting model (BIC=16,176.50), with a high entropy value of 0.96. When final comparisons of the best fitting FA, LCA and FMM models were conducted, the FMM-3 four-class, two-factor model was determined to be the overall best fit for the data. Please see eTables 3–5 in Supplementary Materials for detailed results of the best FA, LCA, and FMM, along with a description of each STiP-5.1 item in eTable 6. Statistically significant positive correlations between the two factors were found for each class (all *p*≤0.037) (see eTable 7 and eFigure 1 in Supplementary Materials for further details). Additionally, the composition of the two factors for each class (as part of the four-class two-factor FMM-3) reflecting self- and interpersonal-functioning can be found in the section including details of the best FMM as in eTable 3 in Supplementary Materials.

**Table 2:** Model fit comparisons for Factor Analysis (FA), Latent Class Analysis (LCA) and Factor Mixture Models (FMM) (*N*=502)

Model	BIC	Entropy
FA		
One-factor	16,695.74	..
Two-factor	16,530.62	..
** Four-factor**	**16,483**.**77**	..
LCA		
One-class	18,384.08	..
Two-class	17,004.65	0.90
Three-class	16,679.33	0.85
Four-class	16,579.73	0.89
Five-class	16,503.81	0.87
Six-class	16,430.33	0.89
Seven-class	16,414.44	0.89
** Eight-class**	**16,402.64**	**0.90**
Nine-class	16,404.63	0.91
FMM		
FMM-1		
Two-class, one-factor	17,004.65	0.90
Three-class, one-factor	16,713.23	0.83
Four-class, one-factor	16,664.62	0.79
Five-class, one-factor	16,657.17	0.72
Six-class, one-factor	16,669.61	0.75
Two-class, two-factor	17,004.65	0.90
Three-class, two-factor	16,662.28	0.84
Four-class, two-factor	16,546.03	0.83
Five-class, two-factor	16,564.68	0.86
Two-class, four-factor	17,004.65	0.90
Three-class, four-factor	16,666.47	0.84
Four-class, four-factor	16,560.03	0.84
Five-class, four-factor	16,591.12	0.86
FMM-2		
Two-class, one-factor	16,654.61	0.45
Three-class, one-factor	16,652.90	0.57
Four-class, one-factor	16,662.92	0.62
Two-class, two-factor	16,418.07	0.58
Three-class, two-factor	16,442.95	0.74
Four-class, two-factor	16,441.68	0.80
Two-class, four-factor	16,220.86	0.84
FMM-3		
Two-class, one-factor	16,465.48	0.97
Three-class, one-factor	16,386.16	0.84
Four-class, one-factor	16,300.85	0.94
Five-class, one-factor	16,275.43	0.89
Six-class, one-factor	16,243.71	0.90
Two-class, two-factor	16,284.33	0.97
Three-class, two-factor	16,265.63	0.92
** Four-class, two-factor**	**16,176.50**	**0.96**
Five-class, two-factor	16,184.15	0.89
Two-class, four-factor	16,222.05	0.85

Items in bold indicate the best model fit for that model type (i.e., FA, LCA, FMM)

### Step 2: Description and characterisation of the best fitting model

**Fig. 1:**
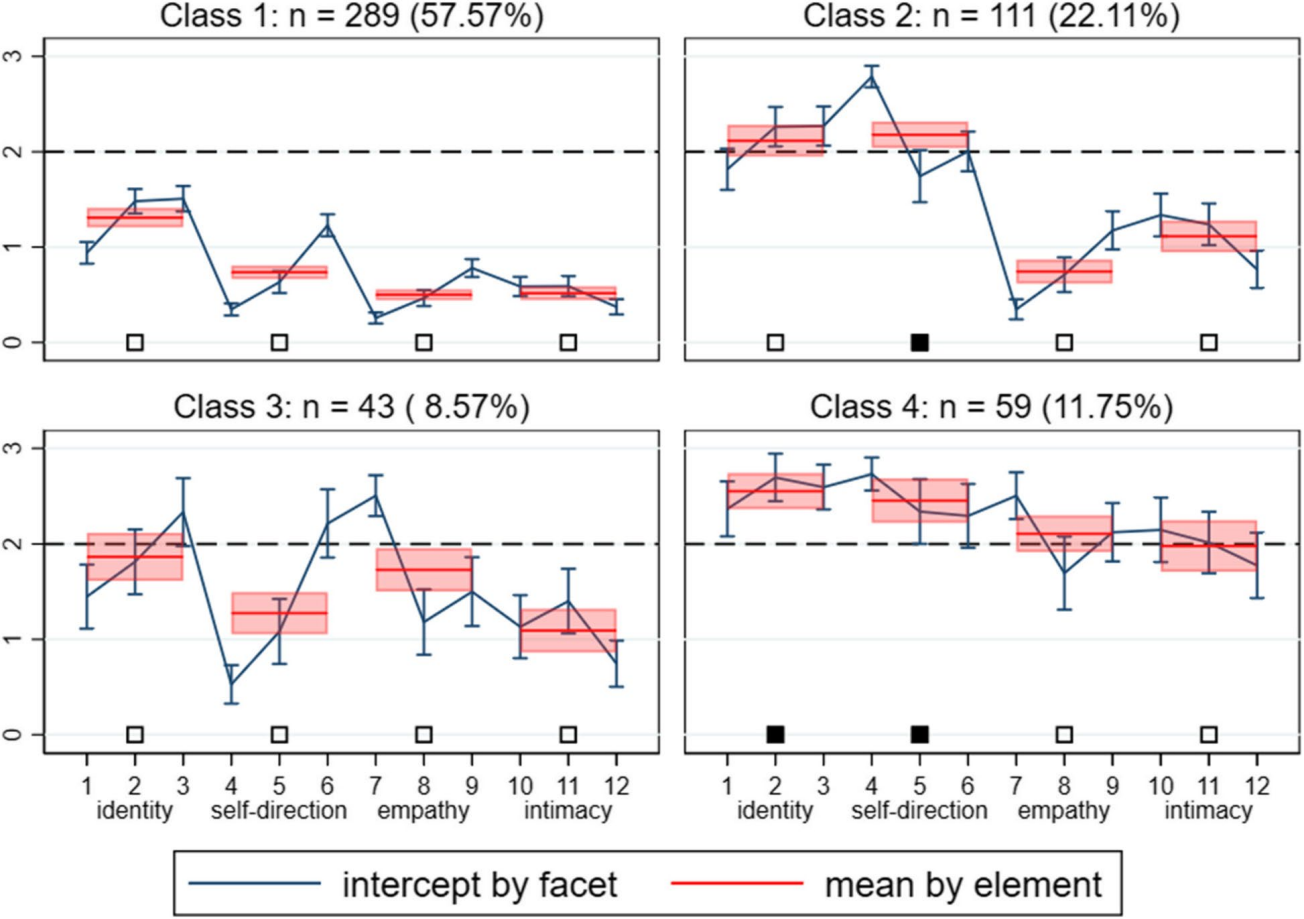
Profiles of the four classes of the best model fit (FMM-3) across STiP-5.1 elements**.** Dashed lines indicate the clinical threshold of ‘moderate or greater’ (2 or higher) impairment (as stipulated in the *DSM-5* AMPD) for each element. Filled black squares indicate reached clinical threshold for this element. Numbers 1–12 indicate the individual STiP-5.1 facets

The four class profiles based on the elements of the STiP-5.1 that were identified by the best fitting overall model (FMM-3, four-class, two-factors) are depicted in Fig. 1. Class 1 comprised the highest number of participants (*n*=289, 57.57%), and did not meet clinically relevant threshold for any of the four elements. Class 2 comprised just over one fifth of the sample (*n*=111, 22.11%) and scored over clinically relevant threshold for the self-direction element, but no others. Class 3 comprised the smallest number of participants (*n*=43, 8.57%) and (like class 1), did not meet clinically relevant threshold for any of the four elements, but appeared to have emerging impairments in identity and empathy. Class 4 comprised 11.75% (*n*= 59) of participants in the sample and was above the clinically relevant threshold for both of the self-functioning elements (identity and self-direction), but not the interpersonal elements – although emerging impairments were evident. Based on their respective scores on the STiP-5.1 elements (i.e., descriptors), the four profiles were labelled as: (1) *Low personality functioning impairment*, (2) *Self-functioning impairment*, (3) *Sub-threshold personality functioning impairment*, and (4) *High personality functioning impairment*. Further detail on the distribution and spread of individual data points in each class profile across self- and interpersonal-functioning (depicting inhomogeneity in each profile), is available in the Supplementary Material (eText 3 and eFigure 1).

Given that neither class 1 nor class 3 met the clinical threshold for any elements of the STiP-5.1, it was of interest to determine whether they significantly differed from one another in this regard. First, the groups were found to be statistically significantly different overall across all elements (χ^2^_(4)_ =124.33,* p* < 0.001). Second, class 3 scored significantly higher on each element compared to class 1: Identity (χ^2^_(1)_ = 15.95, *p* = 0.0001), self-direction (χ^2^_(1)_ = 20.33, *p* < 0.001), empathy (χ^2^_(1)_ = 108.48,* p* < 0.001), and intimacy (χ^2^_(1)_ = 21.76, *p* < 0.001).

Table 3 presents descriptive statistics of demographic and clinical variables across each class profile, as well as for the total sample. Regarding overall STiP-5.1 severity, class 1 was the least severe (*M*=0.8, SD=0.5), class 2 and class 3 were alike in severity (*M*=1.5, SD=0.5; *M*=1.5 SD=0.5, respectively), and class 4 was most severe (*M*=2.3, SD=0.6), meeting diagnostic threshold (over 2) for *general* PD according to *DSM-5* AMPD.

**Table 3:** Descriptive statistics across each class profile and for the total sample

Variable	Class 1 (*n* = 289) 57.57%	Class 2 (*n* = 111) 22.11%	Class 3 (*n* = 43) 8.57%	Class 4 (*n* = 59) 11.75%	Total sample (*N* = 502)
Age (in years)	15.3 (1.5) [15]	15.6 (1.5) [16]	15.4 (1.7) [16.0]	15.4 (1.5) [15.0]	[15.0]
Biological sex					
Female (n, %)	224, 77.5%	94, 84.7%	30, 69.8%	49, 83.1%	-
Male (n, %)	65, 22.5%	17, 15.3%	13, 30.2%	10, 16.9%	-
STiP-5.1 total score	0.8 (0.5) [0.7]	1.5 (0.5) [1.5]	1.5 (0.5) [1.3]	2.3 (0.6) [2.3]	[1.1]
STiP-5.1: Identity	1.3 (0.8) [1.3]	2.1 (0.9) [2.0]	1.9 (0.8) [1.7]	2.5 (0.7) [2.7]	[1.7]
STiP-5.1: Self-direction	0.7 (0.6) [0.7]	2.2 (0.7) [2.0]	1.2 (0.7) [1.3]	2.4 (0.8) [2.3]	[1.2]
STiP-5.1: Empathy	0.5 (0.5) [0.3]	0.7 (0.6) [0.7]	1.7 (0.7) [1.7]	2.1 (0.7) [2.0]	[0.7]
STiP-5.1: Intimacy	0.5 (0.6) [0.3]	1.1 (0.9) [1.0]	1.1 (0.7) [1.0]	2.0 (1.0) [2.0]	[0.7]
Fulfilled BPD criteria	1.9 (1.9) [2.0]	3.6 (2.3) [3.0]	3.3 (2.1) [3.0]	4.4 (2.6) [4.0]	[2.0]
Depressivity (CDRS-R)	46.7 (15.2) [47.0]	60.6 (14.4) [63.0]	51.4 (14.2) [51.0]	60.8 (16.3) [61.0]	[51.0]
Emotion Dysregulation (DERS)	56.2 (13.7) [57.0]	63.7 (10.9) [64.0]	60.5 (13.9) [63.0]	66.3 (10.6) [66.0]	[60.5]
HRQoL (KIDSCREEN-10)	21.1 (6.6) [20.5]	16.1 (5.7) [16.0]	18.1 (5.8) [17.0]	16.5 (6.0) [16.0]	[18.0]
Number of MINI-KID diagnoses	2.2 (1.9) [2.0]	3.6 (2.6) [3.0]	3.4 (2.2) [3.0]	4.0 (2.4) [4.0]	[2.0]
Number of NSSI in the past year	40.4 (67.0) [12.0]	79.4 (110.6) [30.0]	87.2 (131.0) [22.5]	77.9 (107.2) [33.0]	[16.0]
Suicide attempts in the past year	4.0 (32.1) [0.0]	2.9 (8.0) [0.0]	4.3 (15.8) [1.0]	2.7 (5.5) [1.0]	[0.0]

*M (SD)* [Median], *STiP-5.1* Semi-structured Interview for Personality Functioning DSM-5 (higher scores higher impairment), *BPD* Borderline Personality Disorder, *CDRS-R* Children’s Depression Rating Scale – Revised, DERS Difficulties in Emotion Regulation Scale, HRQoL Health Related Quality of Life, *MINI-KID* The Mini International Neuropsychiatric Interview for Children and Adolescents, *NSSI* Non-Suicidal Self-Injury. Means and SD for the total sample are provided in Table 1

**Fig. 2:**
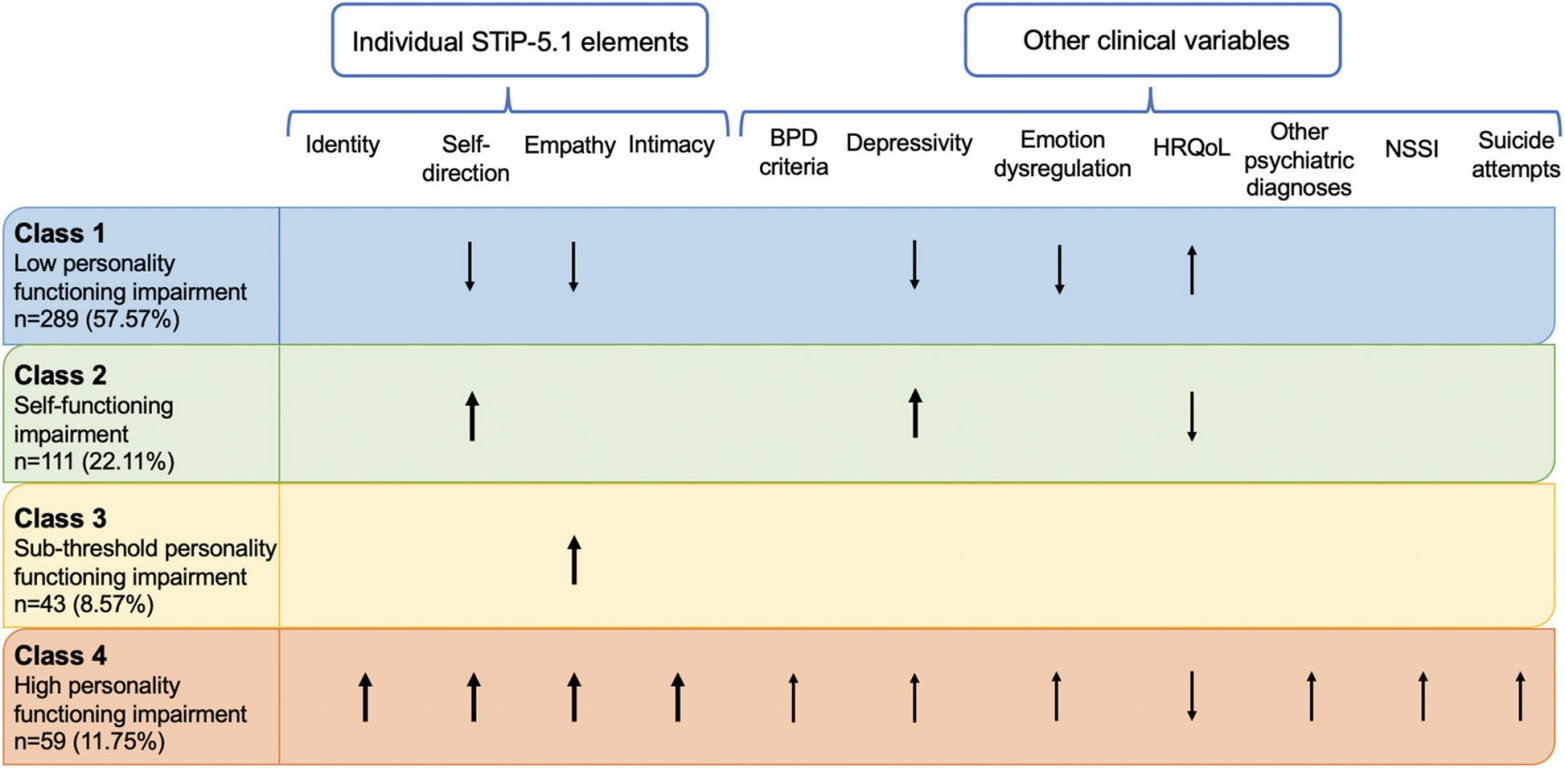
Characterised profiles based on STiP-5.1 elements and other clinical variables as compared to the total sample. ↑=the group median for this variable is above the 60th percentile of the overall sample (i.e., higher than the average). ↓=the group median for this variable is less than the 40th percentile of the overall sample (i.e., lower than the average). Items in bold represent extreme upper or lower scores. In our sample, there are only extreme upper scores. That is, the group median is above the 70th percentile of the overall sample (i.e., they are much higher than the average). Regions without any symbols (blank regions) indicate that the group median for this variable is within the 40th and 60th percentile range of the overall sample (i.e., around average). BPD= Borderline Personality Disorder, HRQoL = Health Related Quality of Life; NSSI=Non-suicidal self-injury. Higher scores on STiP-5.1 elements=higher levels of impairment

When compared with the overall sample, the *low personality functioning impairment* profile (class 1) scored lower than average on self-direction and empathy, but average on identity and intimacy elements on the STiP-5.1. Additionally, they scored lower than average on depressivity and emotion dysregulation, and average for number of psychiatric diagnoses, and NSSI and suicide attempts in the previous year, and scoring higher on HRQoL when compared to the overall sample. The *self-functioning impairment* profile (class 2) had extreme upper scores on self-direction, and scored average for the remainder of the STiP-5.1 elements, in comparison to the overall sample. Notably, they had extreme upper scores in depressivity, but average scores for emotion dysregulation, number of psychiatric diagnoses, NSSI and suicide attempts in the previous year. This class also scored below average on HRQoL. The *sub-threshold personality functioning impairment* profile (class 3) had extreme upper scores on empathy, and scored around average on all other elements and on all remaining clinical variables, when compared to the overall sample. Finally, the *high personality functioning impairment* profile (class 4) had extreme upper scores across all STiP-5.1 elements in comparison to the overall sample, as well as above average scores on all other clinical variables, with the exception of lower than average scores on HRQoL. Refer to Fig. 2 for the clinical profile of each class when compared to the overall sample. Please note that symbols (e.g. arrows regarding ‘higher,’ ‘lower’) in Fig. 2 are based on percentiles derived from median scores of each variable, for each class, relative to the overall sample.

## Discussion

The aims of the current study were to examine the latent structure of a dimensional measure of personality functioning for the first time in a help-seeking sample of adolescents, and to describe clinical profiles emerging from this. Our results showed a complex FMM-3 comprising four-classes and two-factors to be the best model fit, reflecting a hybrid dimensional and categorical model – subgroups with variations along a dimensional severity continuum of personality functioning. Overall, class profiles ranged from a large, relatively unimpaired class [1] to a more severely impaired class [4], with two classes presenting with similar severity, but varying more qualitatively: class 2 had coinciding problems with self-direction and depressivity, whereas class 3 was more mixed, considered ‘at risk’ of fulfilling *general* PD, and showed emerging problems in identity and empathy. Moreover, the two factors of self- and interpersonal-functioning were found to be differently (in strength), but significantly positively related in all classes. In classes where clinically relevant thresholds for individual elements were met (2 and 4), impairments in self-functioning tended to be more pronounced than interpersonal functioning. Our results broadly align with the BPD literature on the search for clusters, in that many studies concluded that discrete classes exist on a severity continuum [29, 31, 32, 34, 35, 46]. Profiles in our study reflect this hybrid of both categorical and dimensional components of personality functioning, confirmed with an FMM (rather than only categorical or only dimensional), albeit, with some further complexity evidenced in the FMM-3.

Of note, while the overall best model comprised a two-factor structure (self- and interpersonal functioning), rather than four factors reflecting each of the elements, examination of each individual element helped to gain important clinical insights into each profile. Therefore, contrary to its conceptual designation as a unidimensional construct of severity, our findings suggest that, while based on a two-factor structure, the four elements can provide nuanced and clinically meaningful information on personality functioning impairments in adolescents, beyond severity alone. This assertion also aligns with specific requirements from individual elements to assign a *specified* PD in the AMPD. Regardless, although debate regarding the factor structure of Criterion A is ongoing [67], the two-factor component reflecting self- and interpersonal functioning, aligns with previous research (mostly in adults) on Criterion A that, despite some mixed results, has mostly found a two-factor structure (resembling self- and interpersonal-functioning) to be most appropriate [26, 68, 69]. Thus, the supporting two-factor component might reflect the broader developmental applicability and suitability of the LPFS in assessing personality pathology across the life course, whereby the structure of self- and interpersonal-functioning is retained in both adult and adolescent samples.

The four profiles partly align with previous work by Gamache and colleagues [47] who, using latent profile analysis (in an adult sample), also identified four unique classes. Although they also included Criterion B of the AMPD with a specific focus on BPD alone, the four classes differed in both severity of impairment in personality functioning and across the elements of Criterion A (as also reflected in the STiP-5.1). Moreover, meaningful qualitative differences could differentiate two profiles at the intermediate level of severity, similarly with our results. In both studies the classes could be differentiated based on the four elements of Criterion A, supporting the assertion that the four elements have individual value in identifying important differences in patients (both adolescents and adults) with personality pathology. Furthermore, our study found that self-functioning impairments coincided with depressivity (class 2), which was also evidenced in a profile in the adult sample of Gamache et al., (2021). One possible explanation for the distinct coinciding problems of self-functioning impairments and depressivity in this group might be cognitive impairments, particularly in cognitive control, often evidenced in individuals with depression [70, 71]. A lack of motivation and apathy in these individuals might impact goal-directed behaviour [71], resulting in a failure to set and achieve developmentally appropriate goals requiring higher level cognitive capacities. Depression affects the very core of a person’s sense of self [72, 73], and identity related distress is strongly associated with depressive symptoms in adolescents [74–76]. Moreover, the development of a sense of identity and goal-directedness are especially important developmental tasks during this period that can be derailed by the emergence of personality pathology [17, 23]. While some identity reconsideration is characteristic of adolescence [77], continued identity uncertainty in emerging adulthood is increasingly associated with depressive symptoms [78], and personality pathology can result from this failure to develop a coherent sense of self [17]. Thus, it is considered a central dimension in personality pathology [76]. Therefore, deficits in self-functioning might be a manifestation of both personality difficulties and depressivity, and may benefit from early intervention.

Given the high rates of comorbidity of PD and depression [79, 80], class 2 might comprise a distinct group of ‘depressive PD,’ [81, 82] which can be suitably captured in Criterion A. In categorical systems, these individuals may have been relegated to the umbrella diagnosis of ‘PD not otherwise specified,’ and Criterion A may provide a more meaningful way to capture their personality difficulties. Regardless, assessment and treatment through the lens of Criterion A can inform clinical management of patients with depression [83, 84].

It is also possible that class 2 reflects the high co-occuring mental disorders common in people with (B)PD [85, 86], given that they presented with the second highest rates in not only depressivity, but also comorbid diagnoses, and emotion dysregulation, the highest overall mean rates of NSSI and suicide attempts, and the lowest overall mean rates of HRQoL, together with the highest overall rates of fulfilled BPD criteria, clinically relevant self-direction issues, and sub-threshold identity issues. It is possible that class 2 represents more common pathways to mental health treatment in people with emerging personality problems. In other words, they might reflect some of the more prevalent reasons for youth presenting to psychiatric services (e.g., general psychological distress, internalizing mental health problems including depression and suicidality [87]).

Some disparities between our findings and those of Gamache et al., (2021), however, are evident, and likely reflect various methodological differences including the use of FMM (instead of latent profile analysis), interview-based assessments of personality pathology (instead of self-reports), the assessment of personality pathology more broadly (instead of strictly BPD) and an adolescent sample (instead of adults). When comparing classes to the overall sample (see Fig. 2), some impairments in interpersonal functioning elements were evident (i.e., extreme upper scores on empathy in classes 3 and 4), probably reflecting the *emerging* clinically relevant empathy impairments in relation to clinical thresholds. However, the majority of the personality functioning impairments in relation to clinically relevant thresholds (see Fig. 1) were centred on self-functioning elements (i.e., heightened identity and self-direction impairment scores in classes 2 and 4), which might therefore be considered as the more defining impairments in this sample. This is in contrast to Gamache et al., (2021), where impairments in empathy were considered to represent a ‘core feature’ of the severe profile in particular. The relative elevations in identity impairments found in our sample (in classes 2, 3, and 4), likely relate to not only an overall disturbance in identity development, but also particular emotion regulation problems, which is captured by one facet in the identity element. Emotion dysregulation has long been considered a core feature of (B)PD [88], and emotion dysregulation scores across all classes in our study exceeded clinically relevant cut-offs (i.e., a score of 43; [89].

Similar to Cavelti and colleagues [30] we found that a more complex FMM-3 was the best fit for our adolescent sample. However, the two-class one-factor solution found in their adolescent sample specific to BPD, was not replicated in our findings of four-classes and two-factors for personality functioning. The four-class solution likely reflects the broader complexity of personality functioning encompassing a more general construct of personality pathology (that is said to cut across all PDs, including BPD), rather than strictly BPD criteria. Indeed, initial work on the STiP-5.1 in adolescents found that the STiP-5.1 did not adequately capture the discrete categorical PD diagnoses in adolescents (unlike in their adult samples), with the exception of BPD. Therefore, it is possible that the STiP-5.1 may have captured some features of BPD (also aligning with the four class solution found by Gamache), with BPD also considered a general marker of severity of personality pathology [14]). Additionally, though, the STiP-5.1 may have also captured other components of personality pathology – not necessarily classic categorical diagnoses – (along with varying severity), that may be more relevant for PD in younger people. This supports the assertion that the STiP-5.1 may be more developmentally suitable for the detection of severity at a young age [20].

Impairments in self-functioning in our study might indeed reflect the developmental maturation processes in adolescence focused on the development of self, highlighting these particular difficulties in those with personality pathology [17]. Simultaneously, the disparities between our findings and Gamache and colleagues potentially reflect the developmental course of personality pathology documented in the literature – that is, more self-focused and impulsive symptoms tend to be more pronounced in adolescence, and interpersonal difficulties come to the fore in adulthood [90]. Aside from this, the *emerging* impairments in empathy in our adolescent sample, especially in class 3 – considered at risk for full-threshold PD – might also reflect the developmental course of PD. Specifically, given that mentalization deficits are posited to form part of the aetiopathogenesis of (B)PD [91, 92], (emerging) impairments in this element might represent a developmental way-station towards more severe PD.

### Clinical implications

One of the major aims of the AMPD was to improve clinical utility and treatment planning [93]. The clinical profiles identified in this study have the potential to assist in clinical decision-making and treatment for personality pathology in young people using: a) a clinical staging approach, detecting the early signs of personality pathology using a severity continuum [90, 94], and b) a modular or targeted treatment approach aligning with emerging dimensional classification models of psychopathology such as the Hierarchical Taxonomy of Psychopathology, (HiTOP; [95], within which Criterion A might be especially suited [96]. For example, although class 1 might not require any intervention for personality pathology, modules aimed at goal setting/attainment, identity difficulties and depressivity may be indicated for class 2. This might be by attending to them simultaneously, but it has been suggested to focus on the underlying personality difficulties, with the addition of multi-modal approaches (e.g., individual and group therapy; [83]. Mentalizing skills for emerging empathy impairments, along with additional psychoeducation and general monitoring of symptom severity for class 3, might be indicated. Mentalizing is highly related to empathy [97, 98] and similar to adults, adolescents with PD may be prone to hypermentalizing [99]. As a possible developmental harbinger for PD, it can manifest later as more pronounced interpersonal difficulties in adulthood, therefore indicating early signs for intervention using mentalization-based treatment techniques [100]. These have promising evidence in adolescents with personality difficulties in improving clinical outcomes [101], thereby potentially preventing the onset of ‘fully manifest’ (or more severe) PD. Modules focused on mentalizing may help improve empathising capacities, and early developments in modular-based treatment for PD are encouraging [102]. The *high personality functioning impairment* profile (class 4), however, likely requires more intensive structured psychological intervention to attend to the broad impairments in personality functioning. For example, psychotherapy is recommended as first-line treatment for PD, and evidence on manualised treatments such as dialectical behavioural therapy for adolescents, mentalization-based therapy, and cognitive-analytic therapy, have some promising findings for adolescents with (B)PD [103–106].

### Strengths and limitations

Strengths of the current study include the large sample size, and that the clinical assessments were conducted by highly trained psychologists, adding to the validity of the data. Additionally, we included a large age range of participants across the span of adolescence (11–18 years of age) providing a broad picture of adolescent personality pathology. However, participants in our study were mostly female (although this is often the case in clinical samples as they tend to be more likely to seek help [107], and therefore results might not be generalizable to populations where males are more represented.

Statistical limitations might include the use of entropy. That is, while it was included to identify over extraction of classes, there is still the potential for some subjects to be misplaced into the incorrect class (even when the entropy value is close to one). Further, we limited statistical comparisons between classes 1 and 3 only, as neither met clinically relevant thresholds for any of the individual elements, and therefore, may have limited interpretation in the absence of statistical comparison between the two. We restricted ourselves to this comparison to avoid introducing multiple testing issues. However, it is possible that other comparisons may have yielded useful information, especially between groups of similar severity. Finally, we focused on Criterion A, and did not incorporate Criterion B traits into the models, which form part of diagnostic criteria in *DSM-5*. This choice to address structural and heterogeneity issues in the dimensional conceptualization of Criterion A was intentionally made based on its alignment with that of the *ICD-11*, and that it more closely reflects the broader (and radical) paradigm shift away from categorical approaches towards dimensional, and therefore it might be similarly applied to the newly established *ICD-11* model. However, given that research in this area (especially in adolescents) is in its infancy, and some debate regarding the utility of dimensional versus trait models is ongoing [108, 109], it would be a worthy endeavour for future work to also incorporate both Criterion A and B into efforts at elucidating latent structure and clinical profiles of personality pathology in young people. Indeed, self-report research on adults in a community sample using clustering based on Criterion B, has revealed a six cluster-solution with some – although, not all – paralleling an increase in Criterion A severity, indicating that Criterion B can be considered (at least to some extent) informative of Criterion A severity in adults [110].

### Future directions

Our findings add to the growing literature on the clinical utility of the AMPD [111], with a focus on Criterion A, and supports dimensional and even transdiagnostic approaches (with the identification of symptom clusters rather than diagnoses) to the understanding of personality pathology, and psychopathology more broadly. Future studies should extend on this by examining these profiles in an even broader transdiagnostic manner, to help integrate evidence regarding not only clinical profiles as manifest symptoms, but also investigating neurobiological underpinnings or phenotypes of such profiles, to elucidate processes of personality pathology across homogenous profiles. Furthermore, given the developmental peculiarities evident across adolescence [17, 112] and the varying manifestations of personality pathology over the life course [113], future research should explore the trajectories of the identified profiles over time to examine their clinical course. Indeed, emerging research using self-report in community studies found four developmental trajectory classes of adolescents regarding impairment in personality functioning, differing in severity, but remaining relatively stable across adolescence [114]. However, it is clear that clinical samples assessed using (semi-) structured interviews are required to enhance this limited evidence base, and to increase validity and clinical utility of the findings.

## Conclusions

Our study provides novel contributions to the ongoing debate regarding the underlying structure of personality pathology, with adolescents differentiated not only quantitatively (dimensionally) but also qualitatively (categorically) across the classes on the STiP-5.1. Therefore, we assert that Criterion A provides more nuanced and clinically relevant information regarding personality pathology in adolescents beyond severity alone (reflected also in the requirements for *specified* PD in the AMPD). That is, in our help-seeking sample, personality functioning comprised a hybrid of both dimensional severity and categorical (qualitative) components, and this perspective, particularly in adolescents, has thus far been largely ignored. This alternative perspective on Criterion A comprising both components in and of itself warrants further research and clinical attention.

### Supplementary Information

**Supplementary Material 1.**

## Data Availability

The datasets generated and/or analysed during the current study are not publicly available as participants did not provide consent for their data to be shared publicly, but are available from the corresponding author on reasonable request.

## Abbreviations

AMPD: Alternative Model of Personality Disorders
BIC: Bayesian Information Criterion
BPD: Borderline Personality Disorder
CDRS-R: The Children’s Depression Rating Scale-Revised
DERS-16: Difficulties in Emotion Regulation Scale
FA: Factor Analysis
FMM: Factor Mixture Models
HiTOP: Hierarchical Taxonomy of Psychopathology
HRQoL: Health-related quality of life
KIDSCREEN-10: The Health-related quality of life questionnaire for children and young people and their parents
LCA: Latent Class Analysis
LPFS: Level of Personality Functioning Scale
MI: Measurement invariance
MINI-KID: The Mini International Neuropsychiatric Interview for Children and Adolescents
NSSI: Non-suicidal self-injury
PD: Personality disorders
SCID-II-PD: Structured Clinical Interview for DSM-IV axis II Personality Disorders
SITBI-G: The Self-Injurious Thoughts and Behaviors Interview German version
STiP-5.1: Semi-Structured interview for Personality Functioning DSM-5
