# Supplementary material for: Clinical profiles of adolescent personality pathology: a latent structure examination of the Semi-Structured Interview for Personality Functioning DSM-5 (STiP-5.1) in a help-seeking sample
Source: Borderline Personal Disord Emot Dysregul. 2024 Apr 9;11:9. doi: 10.1186/s40479-024-00252-5 (PMC11003081; doi:10.1186/s40479-024-00252-5)
Supplement: Supplementary file 1 — Supplementary Material 1. [file 40479_2024_252_MOESM1_ESM.docx]

**Supplementary Material for**

***“Clinical profiles of adolescent personality pathology: A latent structure examination of the Semi-Structured Interview for Personality Functioning DSM-5 (STiP-5.1) in a help-seeking sample.”***

Madelyn Thomson, Marialuisa Cavelti, Stefan Lerch, Julian Koenig, Corinna Reichl, Ines Mürner-Lavanchy, Andrea Wyssen, Michael Kaess

**Contents:** 3 eTexts, 1 eFigure, 7 eTables

- **eText 1:** Brief overview of research elucidating subgroups in (B)PD.
- **eText 2:** Conceptual considerations included in the study
- **eText 3:** Homogeneity in the classes by self- and interpersonal functioning
- **eFigure 1:** Inhomogeneities of each class across self and interpersonal functioning, depicted by individual data points
- **eTable 1**. Specification and graphic illustration of FMM variants. Adapted from Clark et al. (1)
- **eTable** **2**: Additional fit indices for each tested model (N=502)
- **eTable 3:** Composition and item thresholds for the best Factor Mixture Model (FMM-3; 2 classes, four factors) (N=502)
- **eTable 4:** Composition and item thresholds for best factor analysis (FA; 4 factors) (N=502)
- **eTable 5:** Composition of best latent class analysis (LCA; 8 classes) (N=502)
- **eTable 6:** Description of core domains, elements and individual facets (items) with their respective abbreviations
- **eTable 7**: Correlation coefficients between self- and interpersonal-functioning for each class (N=502)

**eText 1:** *Brief overview of research elucidating subgroups in (B)PD.*

Several studies have incorporated variations of cluster-analytic, factor analytic, latent class/trait approaches, and even finite mixture modelling, in an attempt to elucidate meaningful subgroups in categorical BPD. For the most part, only one approach is employed at a time, and many have found a three-cluster solution to best represent subgroups of BPD, often reflecting groups of externalising, externalising, or mixed (“core” BPD) compositions, or combinations of paranoid/schizotypal, and interpersonally unstable (antisocial, aggressive) groups (2–5). Severity dimensions have also been found to help differentiate subgroups (2), and many conclude that discrete classes exist along a severity continuum (6–11). In contrast to the various three-cluster solutions yielded in some studies, others, using FA for example, have found that BPD criteria (including in youth) corresponds mostly to a unidimensional structure (12). When considering BPD in light of the newer conceptualisations of PD (i.e., AMPD and ICD-11), Wolf and colleagues (2) assigned BPD variables to ICD-11 trait and personality functioning domains, and found that a dimensional model with 3 clusters differentiating subtypes by personality traits – roughly corresponding to the model of ICD-11 – was the best fit. However, this was not based on empirical evidence, rather, on case vignettes, limiting its validity in the context of the newer dimensional conceptualisations. When considering personality types more broadly, Rossi and colleagues (13) corroborated a 3-cluster solution of resilient, overcontrolled, and undercontrolled personality types based on DSM-5 Criterion B traits, with differences between groups then based on severity of personality functioning impairment (via self-report). Direct comparison of FA, LCA, and FMM to determine the best structure of PD has also been limited to BPD. One found that a unitary latent trait model was the best fit, and another found a single-factor two-class FMM to be the best fit (14,15).

**eText 2:** *Conceptual considerations included in the study*

Conceptual considerations employed in the study are outlined as follows:

1. In the determination of the number of factors to test. That is, variations of FAs tested were limited to three, following the concept of the STiP-5.1. These variations were based on previous literature, and the logical/conceptual constitution of the instrument and included a one-factor solution (unidimensional) with all facets (items), a two-factor solution capturing self- and interpersonal- functioning, and a 4-factor solution pertaining to each of the four elements. Testing alternatives of 3 or 5 factors, for example, does not reflect the theoretical construction of the instrument and therefore was not included.
2. The composition of each factor (i.e., self- and interpersonal functioning) was determined by the theoretical construction of the instrument. For example, we allowed only for facets associated with self-functioning to comprise self-functioning, and not interpersonal functioning.
3. Although various iterations of FMMs can be constructed with varying degrees of measurement invariance (see below for details), an FMM-4 is considered to be very complex, where factor loadings are allowed to vary between classes, and therefore, have different meaning across each class. This ultimately renders interpretation of the models significantly challenging (if not impossible), thus, limiting its conceptual understanding and clinical applicability. Therefore, we did not iterate FMMs past FMM-3.
4. Numbers of classes or profiles (e.g. for LCA or FMM) should be considered conceptually meaningful and have clinical utility. Therefore, if the best solution determined by statistical procedures contains excessive numbers of classes (i.e., beyond 5 or 6), then alternative options should be considered, as an increase in the number of classes may mean more classes with smaller sizes, and may not make conceptual sense (16).

**eText 3:** *Homogeneity in the classes by self- and interpersonal functioning*

We allowed for inhomogeneity to be present in the clusters, enabling the viewing of individual data point distribution across each profile, as well as the overall means for each profile. For clinical application purposes, each profile cluster was measured against the clinically relevant threshold for impairment in personality functioning (moderate or higher severity) across self- and interpersonal-functioning of the STiP-5.1. This allows for the examination of the cluster profiles based on overall personality pathology and cut across self- and interpersonal functioning. The three-sigma ellipse included in eFigure 1 demonstrates the overlap between classes two and three, but also some of the differences – scores are higher for self-functioning in class 2, but not as high for class 3.

**eFigure 1.** *Inhomogeneities of each class across self and interpersonal functioning, depicted by individual data points*


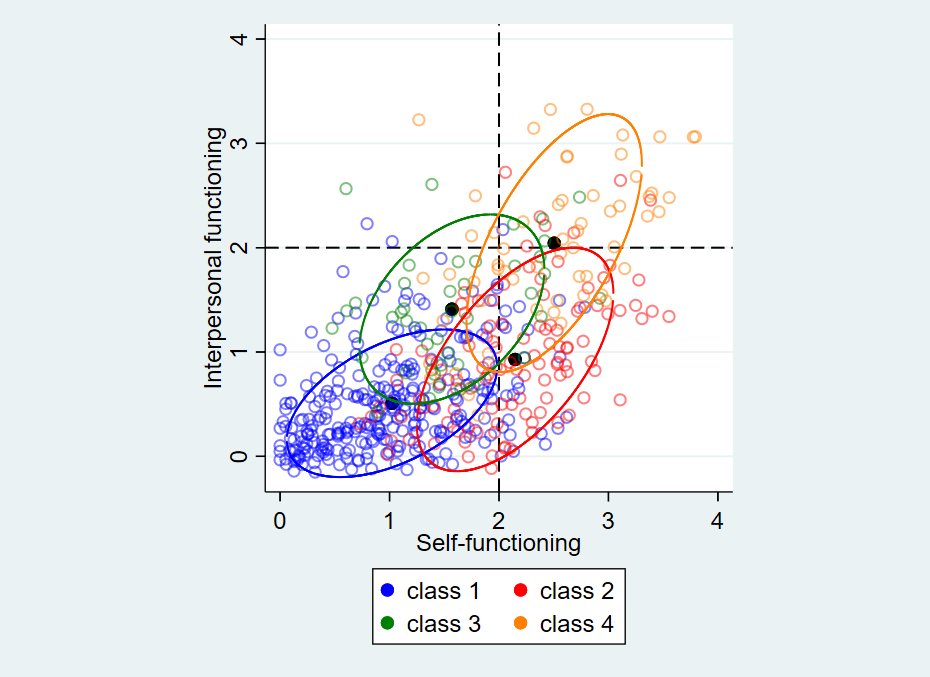


*Notes:* The four black dots reflect the mean of each class across self and interpersonal functioning (i.e., no homogeneity). Larger coloured circles represent the spread of each class. Three sigma ellipses (i.e., inclusion of 99% of the data points are captured in the ellipse) are depicted in the figure.

**eTable 1**. *Specification and graphic illustration of FMM variants. Adapted from Clark et al. (1)*

| FMM Variation | Parameters | | | | | |
| --- | --- | --- | --- | --- | --- | --- |
|  | Factor means | Factor variances and covariances | Item thresholds | Factor loadings | Illustration | Interpretation |
| FMM-1 (latent class factor analytic model) | Class-variant | Fixed to zero | Class-invariant | Class-invariant | 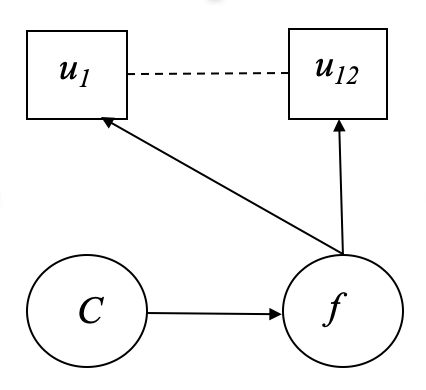 | Most restrictive model. There is no within-class heterogeneity. Class membership is based on each indivdiual’s location on the factor. The factor is measured the same way across classes. |
| FMM-2 (mixture factor analysis) | Class-variant | Class-variant | Class-invariant | Class-invariant | 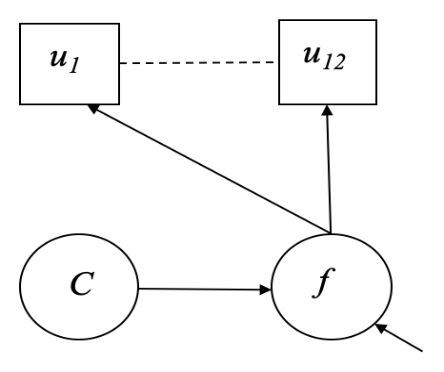 | There is within-class heterogeneity. Class membership is based on each indivdiual’s location on the factor. The factor is measured the same way across classes. |
| FMM-3 | Fixed to zero | Class-variant | Class-variant | Class-invariant | 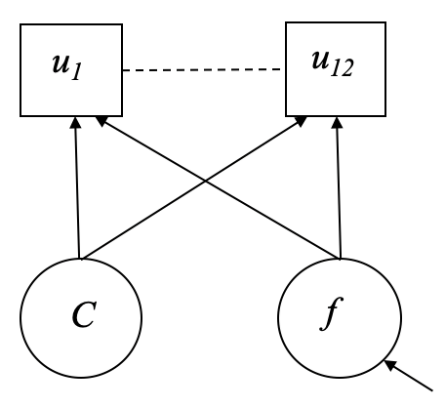 | There are different amounts of heterogeneity within each class. Classes are based on the responses to the items rather than the factor means and variances. |
| FMM-4 | Fixed to zero | Class-variant | Class-variant | Class-variant | 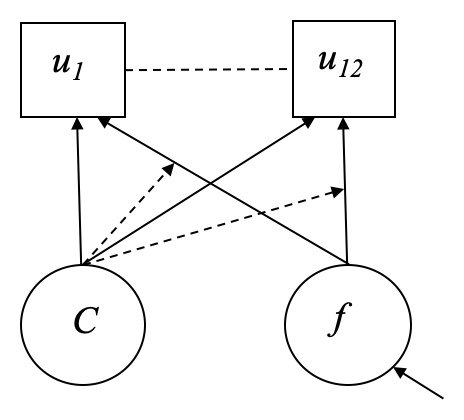 | Least restrictive model. There are different amounts of heterogeneity within each class. Classes are based on the responses to the items rather than the factor means and variances. The factor is not measured the same way across classes, indicating that there is potentially a different factor within each class. |

*Notes. u* = individual STiP-5.1 facets (observed variables), *C* = latent class variable, *f* = latent factor. Boxes indicate observed variables. Circles indicate latent variables. The arrow pointing into the latent factor indicates a residual.

**eTable** **2**: *Additional fit indices for each tested model (N=502)*

| Model | BIC | AIC | saBIC | Entropy | Ek | CLC | ICLBIC |
| --- | --- | --- | --- | --- | --- | --- | --- |
| **fa_4f** | **16483.77** | **16306.59** | **16350.46** | **-** | **-** | **-** | **-** |
| fa_1f | 16695.74 | 16543.87 | 16581.48 | - | - | - | - |
| fa_2f | 16530.62 | 16374.53 | 16413.18 | - | - | - | - |
| lca_9c | 16404.63 | 15864.65 | 15998.35 | 0.91 | 100.37 | 15809.40 | 16605.38 |
| lca_10c | 16415.66 | 15820.83 | 15968.11 | 0.92 | 98.25 | 15735.34 | 16612.16 |
| **lca_8c** | **16402.64** | **15917.50** | **16037.62** | **0.90** | **106.48** | **15900.45** | **16615.59** |
| lca_7c | 16414.44 | 15984.14 | 16090.68 | 0.89 | 104.52 | 15989.18 | 16623.48 |
| lca_6c | 16430.33 | 16054.87 | 16147.84 | 0.89 | 98.04 | 16072.96 | 16626.41 |
| lca_5c | 16503.81 | 16183.20 | 16262.58 | 0.87 | 106.65 | 16244.49 | 16717.11 |
| lca_4c | 16579.73 | 16313.96 | 16379.76 | 0.89 | 76.55 | 16341.06 | 16732.83 |
| lca_3c | 16679.33 | 16468.40 | 16520.62 | 0.85 | 83.83 | 16536.05 | 16846.99 |
| lca_2c | 17004.65 | 16848.56 | 16887.21 | 0.90 | 35.14 | 16844.85 | 17074.94 |
| lca_1c | 18384.08 | 18282.84 | 18307.91 |  |  |  |  |
| **fmm3_4c_2f** | **16176.50** | **15817.92** | **15906.71** | **0.96** | **27.84** | **15703.60** | **16232.18** |
| fmm3_2c_2f | 16284.33 | 16060.74 | 16116.10 | 0.96 | 12.18 | 15979.10 | 16308.69 |
| fmm3_2c_4f | 16222.05 | 15947.84 | 16015.74 | 0.85 | 52.89 | 15923.62 | 16327.83 |
| fmm2_2c_4f | 16220.86 | 16005.71 | 16058.98 | 0.83 | 57.41 | 16018.54 | 16335.69 |
| fmm3_3c_2f | 16265.62 | 15974.54 | 16046.61 | 0.92 | 43.57 | 15923.68 | 16352.76 |
| fmm3_5c_2f | 16184.15 | 15758.07 | 15863.57 | 0.89 | 91.30 | 15738.67 | 16366.75 |
| fmm3_4c_1f | 16300.85 | 15971.80 | 16053.27 | 0.94 | 40.36 | 15896.53 | 16381.58 |
| fmm3_6c_1f | 16243.71 | 15796.53 | 15907.25 | 0.90 | 89.05 | 15762.63 | 16421.80 |
| fmm3_5c_1f | 16275.43 | 15887.32 | 15983.42 | 0.89 | 87.26 | 15877.83 | 16449.94 |
| fmm4_2c_1f | 16432.87 | 16175.54 | 16239.25 | 0.96 | 13.22 | 16079.99 | 16459.32 |
| fmm3_2c_1f | 16465.48 | 16254.55 | 16306.78 | 0.97 | 10.79 | 16176.12 | 16487.05 |
| fmm3_3c_1f | 16386.16 | 16116.17 | 16183.02 | 0.84 | 86.03 | 16160.24 | 16558.23 |
| fmm2_2c_2f | 16418.07 | 16245.11 | 16287.94 | 0.58 | 145.45 | 16454.00 | 16708.97 |
| fmm2_4c_2f | 16441.68 | 16209.66 | 16267.11 | 0.80 | 139.18 | 16378.03 | 16720.05 |
| fmm2_3c_2f | 16442.95 | 16253.11 | 16300.11 | 0.74 | 145.60 | 16454.30 | 16734.14 |
| fmm1_4c_2f | 16546.03 | 16364.63 | 16409.54 | 0.83 | 117.61 | 16513.85 | 16781.25 |
| fmm1_4c_4f | 16560.03 | 16361.75 | 16410.84 | 0.83 | 114.83 | 16497.41 | 16789.68 |
| fmm1_5c_2f | 16564.68 | 16370.62 | 16418.67 | 0.86 | 117.15 | 16512.93 | 16798.98 |
| fmm1_5c_4f | 16591.12 | 16371.75 | 16426.06 | 0.86 | 114.73 | 16497.20 | 16820.57 |
| fmm1_3c_2f | 16662.28 | 16493.54 | 16535.32 | 0.83 | 91.00 | 16595.54 | 16844.28 |
| fmm1_3c_4f | 16666.47 | 16489.29 | 16533.16 | 0.84 | 89.90 | 16585.08 | 16846.26 |
| fmm1_3c_1f | 16713.23 | 16548.71 | 16589.44 | 0.83 | 92.65 | 16656.01 | 16898.54 |
| fmm1_4c_1f | 16664.62 | 16491.65 | 16534.48 | 0.79 | 143.36 | 16696.38 | 16951.34 |
| fmm2_2c_1f | 16654.61 | 16490.08 | 16530.82 | 0.45 | 191.73 | 16795.54 | 17038.06 |
| fmm1_2c_1f | 17004.65 | 16848.56 | 16887.21 | 0.90 | 35.14 | 16844.85 | 17074.94 |
| fmm1_2c_2f | 17004.65 | 16848.56 | 16887.21 | 0.90 | 35.14 | 16844.85 | 17074.94 |
| fmm1_2c_4f | 17004.65 | 16848.56 | 16887.21 | 0.90 | 35.14 | 16844.85 | 17074.94 |
| fmm1_5c_1f | 16657.17 | 16475.77 | 16520.69 | 0.72 | 224.61 | 16838.98 | 17106.38 |
| fmm1_6c_1f | 16669.61 | 16479.77 | 16526.78 | 0.75 | 224.87 | 16839.51 | 17119.34 |
| fmm2_3c_1f | 16652.90 | 16475.72 | 16519.59 | 0.57 | 234.94 | 16861.60 | 17122.79 |
| fmm2_4c_1f | 16662.92 | 16473.09 | 16520.09 | 0.62 | 261.67 | 16906.42 | 17186.26 |

*Notes:* BIC=Baysian Information Criterion; ACI=Akaike Information Criterion; saBIC=sample adjustic Baysian Information Criterion; Ek=unscaled entropy; CLC=Classification Likelihood Criterion; ICLBIC= Integrated classification likelihood BIC approximation; fa=factor analysis; lca=latent class analysis; fmm=factor mixture model; f=factor; c=class. Items in bold indicate the best model of each type (e.g., the best FA, the best LCA, and the best FMM).

**eTable 3:** *Composition and item thresholds for the best Factor Mixture Model (FMM-3; 2 classes, four factors) (N=502)*

| Two-tailed |
| --- |
| Estimate S.E. Est./S.E. P-Value |
| **Latent class 1** |
| F1 by |
| STIP_1_1 1.000 0.000 999.000 999.000 |
| STIP_1_2 1.162 0.092 12.685 0.000 |
| STIP_1_3 1.011 0.104 9.722 0.000 |
| STIP_2_1 0.247 0.048 5.116 0.000 |
| STIP_2_2 0.648 0.102 6.370 0.000 |
| STIP_2_3 0.810 0.110 7.350 0.000 |
| F2 by |
| STIP_3_1 1.000 0.000 999.000 999.000 |
| STIP_3_2 2.665 0.747 3.567 0.000 |
| STIP_3_3 3.026 0.757 3.995 0.000 |
| STIP_4_1 5.480 1.592 3.441 0.001 |
| STIP_4_2 5.525 1.583 3.491 0.000 |
| STIP_4_3 2.830 0.819 3.453 0.001 |
| F2 with |
| F1 0.039 0.012 3.215 0.001 |
| Means |
| F1 0.000 0.000 999.000 999.000 |
| F2 0.000 0.000 999.000 999.000 |
| Intercepts |
| STIP_1_1 0.939 0.058 16.223 0.000 |
| STIP_1_2 1.481 0.066 22.498 0.000 |
| STIP_1_3 1.508 0.068 22.165 0.000 |
| STIP_2_1 0.347 0.032 10.702 0.000 |
| STIP_2_2 0.633 0.059 10.780 0.000 |
| STIP_2_3 1.229 0.059 20.787 0.000 |
| STIP_3_1 0.257 0.030 8.605 0.000 |
| STIP_3_2 0.465 0.042 10.968 0.000 |
| STIP_3_3 0.780 0.048 16.303 0.000 |
| STIP_4_1 0.587 0.051 11.450 0.000 |
| STIP_4_2 0.590 0.054 10.882 0.000 |
| STIP_4_3 0.373 0.041 9.095 0.000 |
| **Latent class 2** |
| F1 by |
| STIP_1_1 1.000 0.000 999.000 999.000 |
| STIP_1_2 1.162 0.092 12.685 0.000 |
| STIP_1_3 1.011 0.104 9.722 0.000 |
| STIP_2_1 0.247 0.048 5.116 0.000 |
| STIP_2_2 0.648 0.102 6.370 0.000 |
| STIP_2_3 0.810 0.110 7.350 0.000 |
| F2 by |
| STIP_3_1 1.000 0.000 999.000 999.000 |
| STIP_3_2 2.665 0.747 3.567 0.000 |
| STIP_3_3 3.026 0.757 3.995 0.000 |
| STIP_4_1 5.480 1.592 3.441 0.001 |
| STIP_4_2 5.525 1.583 3.491 0.000 |
| STIP_4_3 2.830 0.819 3.453 0.001 |
| F2 with |
| F1 0.064 0.023 2.828 0.005 |
| Means |
| F1 0.000 0.000 999.000 999.000 |
| F2 0.000 0.000 999.000 999.000 |
| Intercepts  STIP_1_1 1.816 0.110 16.483 0.000  STIP_1_2 2.262 0.105 21.464 0.000  STIP_1_3 2.270 0.105 21.616 0.000  STIP_2_1 2.788 0.058 48.283 0.000  STIP_2_2 1.744 0.139 12.566 0.000  STIP_2_3 2.004 0.106 18.850 0.000  STIP_3_1 0.347 0.053 6.495 0.000  STIP_3_2 0.710 0.093 7.653 0.000  STIP_3_3 1.175 0.102 11.495 0.000  STIP_4_1 1.337 0.115 11.660 0.000  STIP_4_2 1.238 0.112 11.097 0.000  STIP_4_3 0.765 0.100 7.675 0.000 |
| **Class 3** |
| F1 by |
| STIP_1_1 1.000 0.000 999.000 999.000 |
| STIP_1_2 1.162 0.092 12.685 0.000 |
| STIP_1_3 1.011 0.104 9.722 0.000 |
| STIP_2_1 0.247 0.048 5.116 0.000 |
| STIP_2_2 0.648 0.102 6.370 0.000 |
| STIP_2_3 0.810 0.110 7.350 0.000 |
| F2 by |
| STIP_3_1 1.000 0.000 999.000 999.000 |
| STIP_3_2 2.665 0.747 3.567 0.000 |
| STIP_3_3 3.026 0.757 3.995 0.000 |
| STIP_4_1 5.480 1.592 3.441 0.001 |
| STIP_4_2 5.525 1.583 3.491 0.000 |
| STIP_4_3 2.830 0.819 3.453 0.001 |
| F2 with |
| F1 0.035 0.020 1.729 0.084 |
| Means |
| F1 0.000 0.000 999.000 999.000 |
| F2 0.000 0.000 999.000 999.000  Intercepts  STIP_1_1 1.448 0.171 8.466 0.000  STIP_1_2 1.813 0.173 10.457 0.000  STIP_1_3 2.335 0.182 12.837 0.000  STIP_2_1 0.528 0.103 5.133 0.000  STIP_2_2 1.083 0.174 6.219 0.000  STIP_2_3 2.215 0.183 12.133 0.000  STIP_3_1 2.507 0.109 22.986 0.000  STIP_3_2 1.182 0.175 6.745 0.000  STIP_3_3 1.500 0.184 8.166 0.000  STIP_4_1 1.132 0.169 6.707 0.000  STIP_4_2 1.401 0.173 8.121 0.000  STIP_4_3 0.745 0.124 6.013 0.000 |
| **Class 4** |
| F1 by |
| STIP_1_1 1.000 0.000 999.000 999.000 |
| STIP_1_2 1.162 0.092 12.685 0.000 |
| STIP_1_3 1.011 0.104 9.722 0.000 |
| STIP_2_1 0.247 0.048 5.116 0.000 |
| STIP_2_2 0.648 0.102 6.370 0.000 |
| STIP_2_3 0.810 0.110 7.350 0.000 |
| F2 by |
| STIP_3_1 1.000 0.000 999.000 999.000 |
| STIP_3_2 2.665 0.747 3.567 0.000 |
| STIP_3_3 3.026 0.757 3.995 0.000 |
| STIP_4_1 5.480 1.592 3.441 0.001 |
| STIP_4_2 5.525 1.583 3.491 0.000 |
| STIP_4_3 2.830 0.819 3.453 0.001 |
| F2 with |
| F1 0.066 0.027 2.428 0.015 |
| Means |
| F1 0.000 0.000 999.000 999.000 |
| F2 0.000 0.000 999.000 999.000  Intercepts  STIP_1_1 2.368 0.147 16.081 0.000  STIP_1_2 2.697 0.127 21.319 0.000  STIP_1_3 2.596 0.119 21.810 0.000  STIP_2_1 2.732 0.087 31.307 0.000  STIP_2_2 2.340 0.173 13.514 0.000  STIP_2_3 2.295 0.171 13.439 0.000  STIP_3_1 2.506 0.124 20.137 0.000  STIP_3_2 1.695 0.196 8.662 0.000  STIP_3_3 2.123 0.156 13.604 0.000  STIP_4_1 2.148 0.173 12.441 0.000  STIP_4_2 2.015 0.164 12.253 0.000  STIP_4_3 1.775 0.175 10.140 0.000 |

*Notes:* S.E.=standard error; Est.= estimate. For a full description of each variable, please refer to eTable 6.

**eTable 4:** *Composition and item thresholds for best factor analysis (FA; 4 factors) (N=502)*

| Two-Tailed |
| --- |
| Estimate S.E. Est./S.E. P-Value |
| F1 by |
| STIP_1_1 1.000 0.000 999.000 999.000 |
| STIP_1_2 1.021 0.064 15.838 0.000 |
| STIP_1_3 0.885 0.067 13.154 0.000 |
| F2 by |
| STIP_2_1 1.000 0.000 999.000 999.000 |
| STIP_2_2 1.012 0.083 12.148 0.000 |
| STIP_2_3 0.862 0.079 10.913 0.000 |
| F3 by |
| STIP_3_1 1.000 0.000 999.000 999.000 |
| STIP_3_2 0.881 0.081 10.936 0.000 |
| STIP_3_3 1.020 0.088 11.579 0.000 |
| F4 by |
| STIP_4_1 1.000 0.000 999.000 999.000 |
| STIP_4_2 0.992 0.057 17.441 0.000 |
| STIP_4_3 0.611 0.051 11.970 0.000 |
| F1 with |
| F2 0.606 0.063 9.656 0.000 |
| F3 0.345 0.044 7.894 0.000 |
| F4 0.520 0.055 9.396 0.000 |
| F2 with |
| F3 0.398 0.048 8.315 0.000 |
| F4 0.503 0.058 8.631 0.000 |
| F3 with |
| F4 0.469 0.050 9.475 0.000 |
| Intercepts |
| STIP_1_1 1.339 0.050 26.544 0.000 |
| STIP_1_2 1.821 0.051 35.474 0.000 |
| STIP_1_3 1.871 0.053 35.629 0.000 |
| STIP_2_1 1.169 0.056 21.037 0.000 |
| STIP_2_2 1.112 0.057 19.513 0.000 |
| STIP_2_3 1.606 0.051 31.714 0.000 |
| STIP_3_1 0.731 0.046 15.931 0.000 |
| STIP_3_2 0.723 0.042 17.295 0.000 |
| STIP_3_3 1.084 0.045 23.983 0.000 |
| STIP_4_1 0.978 0.050 19.644 0.000 |
| STIP_4_2 0.966 0.050 19.471 0.000 |
| STIP_4_3 0.653 0.042 15.642 0.000 |

*Notes:* S.E.=standard error; Est.= estimate. For a full description of each item, please refer to eTable 6.

**eTable 5:** *Composition of best latent class analysis (LCA; 8 classes) (N=502)*

| Two-Tailed  Estimate S.E. Est./S.E. P-Value |
| --- |
| **Latent Class 1** |
| Means |
| STIP_1_1 1.189 0.193 6.149 0.000 |
| STIP_1_2 1.552 0.222 6.980 0.000 |
| STIP_1_3 2.232 0.240 9.286 0.000 |
| STIP_2_1 0.590 0.169 3.487 0.000 |
| STIP_2_2 1.044 0.211 4.942 0.000 |
| STIP_2_3 2.159 0.257 8.396 0.000 |
| STIP_3_1 2.555 0.210 12.193 0.000 |
| STIP_3_2 1.160 0.283 4.098 0.000 |
| STIP_3_3 1.482 0.275 5.379 0.000 |
| STIP_4_1 0.879 0.181 4.862 0.000 |
| STIP_4_2 1.069 0.226 4.720 0.000 |
| STIP_4_3 0.685 0.166 4.113 0.000 |
| **Latent Class 2** |
| Means |
| STIP_1_1 1.512 0.259 5.834 0.000 |
| STIP_1_2 2.373 0.218 10.887 0.000 |
| STIP_1_3 2.330 0.299 7.806 0.000 |
| STIP_2_1 0.583 0.132 4.427 0.000 |
| STIP_2_2 0.925 0.248 3.730 0.000 |
| STIP_2_3 1.857 0.286 6.493 0.000 |
| STIP_3_1 0.293 0.079 3.704 0.000 |
| STIP_3_2 0.547 0.232 2.355 0.019 |
| STIP_3_3 0.925 0.198 4.676 0.000 |
| STIP_4_1 0.617 0.184 3.355 0.001 |
| STIP_4_2 0.579 0.155 3.727 0.000 |
| STIP_4_3 0.471 0.132 3.567 0.000 |
| **Latent Class 3** |
| Means |
| STIP_1_1 0.511 0.111 4.627 0.000 |
| STIP_1_2 0.837 0.152 5.513 0.000 |
| STIP_1_3 0.890 0.129 6.918 0.000 |
| STIP_2_1 0.194 0.050 3.911 0.000 |
| STIP_2_2 0.411 0.071 5.826 0.000 |
| STIP_2_3 0.780 0.080 9.755 0.000 |
| STIP_3_1 0.221 0.041 5.450 0.000 |
| STIP_3_2 0.269 0.048 5.561 0.000 |
| STIP_3_3 0.518 0.050 10.450 0.000 |
| STIP_4_1 0.305 0.053 5.730 0.000 |
| STIP_4_2 0.286 0.053 5.448 0.000 |
| STIP_4_3 0.246 0.055 4.467 0.000 |
| **Latent Class 4** |
| Means |
| STIP_1_1 1.279 0.193 6.628 0.000 |
| STIP_1_2 1.817 0.156 11.610 0.000 |
| STIP_1_3 1.929 0.184 10.479 0.000 |
| STIP_2_1 2.800 0.086 32.419 0.000 |
| STIP_2_2 1.463 0.182 8.020 0.000 |
| STIP_2_3 1.711 0.159 10.762 0.000 |
| STIP_3_1 0.418 0.108 3.859 0.000 |
| STIP_3_2 0.524 0.095 5.499 0.000 |
| STIP_3_3 0.887 0.121 7.341 0.000 |
| STIP_4_1 0.685 0.124 5.502 0.000 |
| STIP_4_2 0.640 0.131 4.892 0.000 |
| STIP_4_3 0.368 0.122 3.001 0.003 |
| **Latent Class 5** |
| Means |
| STIP_1_1 2.818 0.238 11.815 0.000 |
| STIP_1_2 3.313 0.346 9.588 0.000 |
| STIP_1_3 3.049 0.154 19.774 0.000 |
| STIP_2_1 2.724 0.530 5.137 0.000 |
| STIP_2_2 2.227 0.601 3.703 0.000 |
| STIP_2_3 2.163 0.344 6.293 0.000 |
| STIP_3_1 0.142 0.185 0.770 0.442 |
| STIP_3_2 0.872 0.218 3.993 0.000 |
| STIP_3_3 1.338 0.245 5.452 0.000 |
| STIP_4_1 2.208 0.719 3.072 0.002 |
| STIP_4_2 1.881 0.317 5.932 0.000 |
| STIP_4_3 1.147 0.228 5.020 0.000 |
| **Latent Class 6** |
| Means |
| STIP_1_1 1.524 0.562 2.709 0.007 |
| STIP_1_2 2.218 0.308 7.208 0.000 |
| STIP_1_3 2.358 0.348 6.771 0.000 |
| STIP_2_1 0.378 0.376 1.004 0.315 |
| STIP_2_2 0.806 0.695 1.159 0.247 |
| STIP_2_3 1.761 0.379 4.646 0.000 |
| STIP_3_1 0.652 0.272 2.401 0.016 |
| STIP_3_2 1.264 0.631 2.002 0.045 |
| STIP_3_3 1.721 0.338 5.089 0.000 |
| STIP_4_1 2.110 0.668 3.160 0.002 |
| STIP_4_2 2.518 0.856 2.941 0.003 |
| STIP_4_3 0.786 0.371 2.117 0.034 |
| **Latent Class 7** |
| Means |
| STIP_1_1 2.940 0.200 14.693 0.000 |
| STIP_1_2 3.279 0.174 18.846 0.000 |
| STIP_1_3 2.989 0.155 19.333 0.000 |
| STIP_2_1 2.897 0.145 19.965 0.000 |
| STIP_2_2 3.048 0.204 14.978 0.000 |
| STIP_2_3 2.678 0.237 1.296 0.000 |
| STIP_3_1 2.732 0.184 14.812 0.000 |
| STIP_3_2 2.475 0.228 10.848 0.000 |
| STIP_3_3 2.415 0.221 10.949 0.000 |
| STIP_4_1 2.890 0.172 16.823 0.000 |
| STIP_4_2 2.843 0.181 15.677 0.000 |
| STIP_4_3 2.571 0.184 13.950 0.000 |
| **Latent Class 8** |
| Means |
| STIP_1_1 2.144 0.181 11.850 0.000 |
| STIP_1_2 2.318 0.220 10.559 0.000 |
| STIP_1_3 2.341 0.250 9.359 0.000 |
| STIP_2_1 2.435 0.333 7.302 0.000 |
| STIP_2_2 1.903 0.254 7.497 0.000 |
| STIP_2_3 2.426 0.180 13.504 0.000 |
| STIP_3_1 1.940 0.355 5.466 0.000 |
| STIP_3_2 1.265 0.243 5.215 0.000 |
| STIP_3_3 2.151 0.190 11.320 0.000 |
| STIP_4_1 2.038 0.363 5.611 0.000 |
| STIP_4_2 1.972 0.308 6.394 0.000 |
| STIP_4_3 1.408 0.251 5.612 0.000 |

*Notes:* S.E.=standard error; Est.= estimate. For a full description of each variable, please refer to eTable 6.

**eTable 6:** *Description of core domains, elements and individual facets (items) with their respective abbreviations*

| **Core domains** | **Elements** | **Facets** |
| --- | --- | --- |
| Self-functioning | Identity | **STIP_1_1:** Experience of oneself as unique, with clear boundaries between self and others |
|  |  | **STIP_1_2:** Stability of self-esteem and accuracy of self-appraisal |
|  |  | **STIP_1_3:** Capacity for, and ability to regulate, a range of emotional experience |
|  | Self-direction | **STIP_2_1:** Pursuit of coherent and meaningful short-term and life goals |
|  |  | **STIP_2_2:** Utilization of constructive and prosocial internal standards of behaviour |
|  |  | **STIP_2_3:** Ability to self-direct productively |
| Interpersonal functioning | Empathy | **STIP_3_1:** Comprehension and appreciation of others’ experiences and motivations |
|  |  | **STIP_3_2:** Tolerance of differing perspectives |
|  |  | **STIP_3_3:** Understanding the effects of one’s own behaviour on others |
|  | Intimacy | **STIP_4_1:** Depth and duration of connection with others |
|  |  | **STIP_4_2:** Desire and capacity for closeness |
|  |  | **STIP_4_3:** Mutuality of regard as reflected in interpersonal behaviour |

*Note:* Abbreviated items correspond to items in eTables 3 - 5.

**eTable 7**: *Correlation coefficients between self- and interpersonal-functioning for each class (N=502)*

| Class | Coefficient | Standard error | z | P>\|z\| | [95% conf. interval] | |
| --- | --- | --- | --- | --- | --- | --- |
| 1 | .5429775 | .083123 | 6.53 | 0.000 | .3800594 | .7058956 |
| 2 | .6542887 | .1048907 | 6.24 | 0.000 | .4487067 | .8598707 |
| 3 | .410771 | .1972057 | 2.08 | 0.037 | .0242549 | .797287 |
| 4 | .646686 | .1408482 | 4.59 | 0.000 | .3706287 | .9227433 |

**References for Supplementary Materials**

1. Clark SL, Muthén B, Kaprio J, D’Onofrio BM, Viken R, Rose RJ. Models and Strategies for Factor Mixture Analysis: An Example Concerning the Structure Underlying Psychological Disorders. Struct Equ Model Multidiscip J. 2013 Oct 1;20(4).

2. Wolf K, Scharoba J, Noack R, Keller A, Weidner K. Subtypes of borderline personality disorder in a day-clinic setting-Clinical and therapeutic differences. Personal Disord. 2023 Sep;14(5):555–66.

3. Smits ML, Feenstra DJ, Bales DL, de Vos J, Lucas Z, Verheul R, et al. Subtypes of borderline personality disorder patients: a cluster-analytic approach. Borderline Personal Disord Emot Dysregulation. 2017 Jul 3;4(1):16.

4. Antoine SM, Fredborg BK, Streiner D, Guimond T, Dixon-Gordon KL, Chapman AL, et al. Subgroups of borderline personality disorder: A latent class analysis. Psychiatry Res. 2023 May 1;323:115131.

5. Lenzenweger MF, Clarkin JF, Yeomans FE, Kernberg OF, Levy KN. Refining the borderline personality disorder phenotype through finite mixture modeling: implications for classification. J Personal Disord. 2008 Aug;22(4):313–31.

6. Bornovalova MA, Levy R, Gratz KL, Lejuez CW. Understanding the Heterogeneity of BPD Symptoms through Latent Class Analysis: Initial Results and Clinical Correlates among Inner-city Substance Users. Psychol Assess. 2010 Jun;22(2):233–45.

7. Clifton A, Pilkonis PA. Evidence for a single latent class of Diagnostic and Statistical Manual of Mental Disorders borderline personality pathology. Compr Psychiatry. 2007 Feb;48(1):70–8.

8. Fossati A, Maffei C, Bagnato M, Donati D, Namia C, Novella L. Latent structure analysis of DSM-IV borderline personality disorder criteria. Compr Psychiatry. 1999 Feb;40(1):72–9.

9. Shevlin M, Dorahy M, Adamson G, Murphy J. Subtypes of borderline personality disorder, associated clinical disorders and stressful life-events: a latent class analysis based on the British Psychiatric Morbidity Survey. Br J Clin Psychol. 2007 Sep;46(Pt 3):273–81.

10. Slavin-Stewart C, Boylan K, Burke JD. Subgroups of Adolescent Girls With Borderline Personality Disorder Symptoms. J Personal Disord. 2018 Oct;32(5):636–53.

11. Oladottir K, Wolf-Arehult M, Ramklint M, Isaksson M. Cluster analysis of personality traits in psychiatric patients with borderline personality disorder. Borderline Personal Disord Emot Dysregulation. 2022 Feb 8;9(1):7.

12. Michonski JD. The Underlying Factor Structure of DSM criteria in Youth BPD. In: Sharp C, Tackett JL, editors. Handbook of Borderline Personality Disorder in Children and Adolescents [Internet]. New York, NY: Springer; 2014 [cited 2021 Jan 4]. p. 35–48. Available from: https://doi.org/10.1007/978-1-4939-0591-1_4

13. Rossi G, Weekers LC, Hutsebaut J. Resilient, undercontrolled, and overcontrolled personality types based upon DSM-5 maladaptive personality traits. Heliyon. 2021 May 1;7(5):e06938.

14. Conway C, Hammen C, Brennan P. A Comparison of Latent Class, Latent Trait, and Factor Mixture Models of DSM-IV Borderline Personality Disorder Criteria in a Community Setting: Implications for DSM-5. J Personal Disord. 2012 Oct 1;26(5):793–803.

15. Hallquist MN, Pilkonis PA. Refining the phenotype of borderline personality disorder: Diagnostic criteria and beyond. Personal Disord. 2012 Jul;3(3):228–46.

16. Weller BE, Bowen NK, Faubert SJ. Latent Class Analysis: A Guide to Best Practice. J Black Psychol. 2020 May 1;46(4):287–311.
